# Re-envisioning the rotating medical student anatomic pathology educational experience: integration of asynchronous histology modular content with clinical service work under a fluid rotation model

**DOI:** 10.1016/j.acpath.2025.100225

**Published:** 2025-10-07

**Authors:** Alex P. Tannenbaum, Paul S. Weisman, Jessica Gulliver, Ryan Sappenfield, Qiong Zhang, Mark Sharobim, Ande R. Marchini, Colleen Alexander, Shelly Cook, Levi Endelman, Qinyuan Li, Kami Elzinga, Andrey Prilutskiy, Rong Hu, Jefree J. Schulte, Margarita Consing-Gangelhoff, Catherine Bodnar, Claire Castaneda, Erin G. Brooks

**Affiliations:** a University of Wisconsin Hospitals and Clinics, Madison, WI, USA; b Department of Pathology and Laboratory Medicine, University of Wisconsin-Madison, Madison, WI, USA

**Keywords:** Clinical clerkship, Education, Histology, Medical education, Pathology, Undergraduate medical education

## Abstract

With the modern paradigm shift toward an integrated, organ-systems-based structure, United States medical students now receive limited formal training in histology. This places additional strain on pathology departments and assigned-resident educators during medical student clinical clerkships, as they must focus more time on basic principles of histology instead of the cases at hand. Schools have explored the use of asynchronous modular learning resources to build and support histology education outside of the traditional didactic settings, but the successful integration of modular content with traditional pathology rotations has not yet been fully explored. This survey-based study aims to evaluate the effectiveness of a newly implemented anatomic pathology curriculum that integrates asynchronous modular histology content, created in-house by department residents and faculty, with service-based pathology rotations. Postrotation surveys of medical student learners (n = 31/35, 89%), using a 10-point Likert scale, gathered across the 2024–2025 academic year, revealed high ratings for rotation organization, usefulness, histology education, module experience, and rotation personalization. Free-response feedback themes from learners reinforced the value of the integrated educational experience and helpfulness of the modular content. Postrotation surveys of residents assigned to these students (n = 7/14, 50%) revealed a better balance between management of daily workloads and the educational experience of their learners. Faculty surveys (n = 10) revealed a high perceived usefulness of the new curricular model. In all, implementation of this novel curriculum has been effective and popular with rotating students, residents, and faculty at our institution and could likewise serve as an effective model for others.

## Introduction

Histology is a cornerstone of didactic medical education, providing medical students with foundational knowledge of physiology and disease pathophysiology. Traditionally, histology education was structured as a standalone course during the first two years of medical school. As medical curricula have evolved in recent years, however, there has been a paradigm shift toward an organ-systems-based structure,^1^ with histology instruction being partially or fully integrated into other preclinical basic science courses.^2^ This transition has led to reductions in the time dedicated to histology and pathobiology education.^3^, ^4^, ^5^ As a result, US medical students now receive limited formal training in histology, which has led to a noticeable decline in student ability to understand even basic histologic concepts.^5^

At our institution, the 2016 implementation of the ForWard Curriculum resulted in the restructuring of medical education into three phases with increased emphasis on early clinical exposure and integration of basic sciences. While pathology remains threaded throughout the curriculum, the shortened preclinical phase (i.e. reduced from 24 mo to 18 mo) and loss of dedicated pathology and histology courses necessarily reduced students' histopathology exposure to occasional lectures and case-based learning sessions. Consequently, students have limited opportunities to attain a thorough grounding in histopathology fundamentals, which may reduce their interest and readiness for the field. Most dedicated pathology experiences occur late in the curriculum as electives, often after students have chosen other specialties as career paths.

This gap in histology education has placed additional strain on pathology departments during medical student clinical clerkships. At our institution, residents and faculty often need to dedicate substantial time to teaching basic histology concepts during case preview and sign-out in the various anatomic subspecialties. This necessarily detracts from the educator's ability to focus on teaching the more complex, nuanced aspects of the cases. At higher-volume academic institutions in general, the imbalance between educational responsibilities and clinical duties can be particularly pronounced, as case volumes are rising^6^, ^7^, ^8^ and time dedicated to basic teaching while on service is commensurately reduced. This imbalance may create a challenging relationship, as the higher the resident and faculty educator caseload, the more it potentially detracts from the medical student educational experience.

Higher-level concepts in histology that crop up while rotating on an anatomic pathology (AP) service or during pathology-focused educational activities can be perceived as overwhelming, confusing, or irrelevant to the student's future career path if the proper educational foundation is not provided.^9^, ^10^, ^11^ The overall lack of histology exposure, coupled with these negative feelings, has potentially detrimental effects on the ability of the specialty to recruit new trainees and/or generate interest among learners.^12^, ^13^, ^14^, ^15^ Moreover, the inability to convey a pathology experience reflective of actual practice can impact students' understanding of the utility and limitations of the field, potentially leading to long-term challenges with interdisciplinary collaboration in their future clinical roles.^16^

Pathology departments often have limited influence over the integrated curricula of medical schools outside of dedicated pathology clerkships.^17^ To address these educational gaps, while remaining cognizant of curricular restrictions, some schools have explored the use of asynchronous modular learning resources to build and support histology education outside of the traditional didactic settings.^18^, ^19^, ^20^, ^21^ This model gained popularity as a result of social distancing measures during the COVID-19 pandemic and has since been embraced and expanded upon by medical educators.^22^, ^23^, ^24^ These modular resources have shown potential for enhancing students' understanding of basic histology but fall short of conveying the practical aspects of a service-based pathology clerkship. Successful integration of modular content with traditional pathology rotations has not yet been fully explored; this kind of integrated approach has the potential to provide students with basic histology education while also offering the opportunity to apply this knowledge in a hands-on clinical context. Further, having activities for students that can be employed at flexible times and do not require the presence or attention of residents or faculty can provide more flexibility for educators to balance their service responsibilities while maintaining quality educational experiences for their learners.

This study aims to evaluate the effectiveness of a newly implemented AP curriculum that integrates asynchronous modular histology content with service-based pathology rotations. The intent of this integrated approach is to provide a strong histology background for rotating medical students, thereby increasing the educational value students receive from their rotation service work, while providing a better balance of educational and service responsibilities for residents and faculty educators.

## Materials and methods

### Study population

Learners included University of Wisconsin School of Medicine and Public Health senior medical students (third- and fourth-year students) on two- to four-week-long elective rotations in pathology, as well as visiting medical students from other United States medical schools, during the 2024–2025 academic year. Medical student learners were surveyed following rotations in which histology comprised a significant portion of the rotation experience at our institution (e.g. surgical pathology and autopsy). Residents who had prior experience with a learner on the former curriculum were surveyed when they had a learner enrolled in this new curricular model assigned to their service. Faculty members covering the autopsy and surgical pathology subspecialties were selected to receive the survey if they had continuous supervisory responsibilities for learners assigned to their service during the 2024–2025 academic year.

### Curriculum design

A re-envisioned rotation experience was designed wherein expectations were divided between the categories of “independent study” and “service work.” “Independent study” included student completion of a series of in-house, interactive, asynchronous modules on topics covering basic histopathology. Please see the “module design” section for further details on the modular content. Learners who rotated on surgical pathology for ≥1 wk at a stretch were required to complete the modular content associated with their assigned subspecialty service within that timeframe. Medical students rotating on autopsy for ≥1 wk at a stretch were likewise encouraged to complete the histology modules (service work permitting.) Self-assessment quizzes were constructed for each curricular module and assigned to learners accordingly. Additionally, learners were provided with a list of other resources, including textbook recommendations and online educational websites, for use of self-study.

**Fig. 1:**
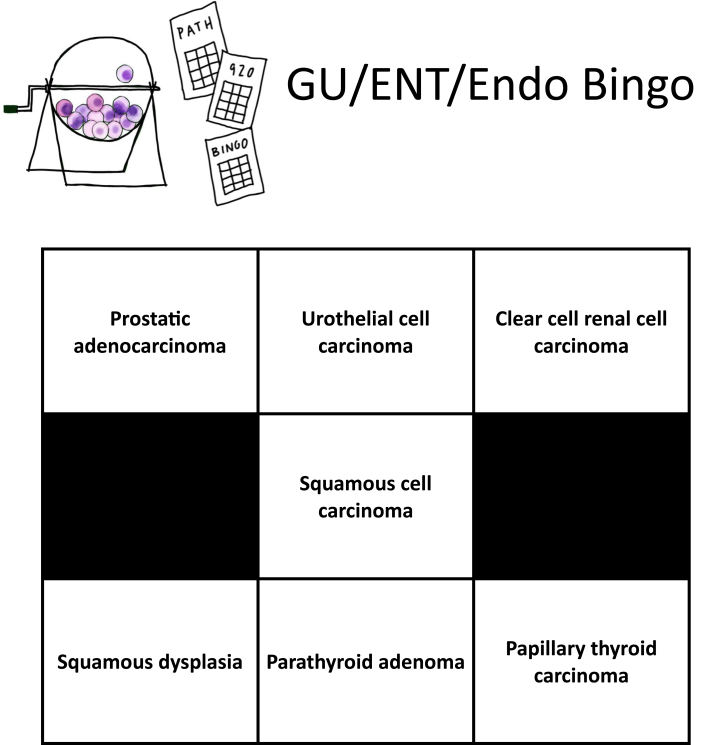
An example of a modified bingo card for the genitourinary, head and neck, and endocrine service.

“Service work” included standard rotation expectations, i.e. previewing cases with residents, signing out cases with faculty, observing frozen sections and grossing, assisting with autopsy prosections, and attending daily quality assurance conferences. In addition, a modified bingo card containing 7–11 of the most common “bread-and-butter” entities for the particular subspecialty was provided to the student as a tool to help guide learning toward the must-know pathologies. Hard copies of the subspecialty-appropriate bingo card were distributed to each student at the beginning of their rotation week. An example of a modified bingo card is presented in Fig. 1. Additionally, our department adopted a more fluid rotation model; learners were provided with a comprehensive schedule of all daily and weekly ongoing educational activities across both anatomic and clinical pathology. Residents and faculty were encouraged to inquire about their students' interests and help guide them toward corresponding “high yield” activities. Once an activity of interest was identified, learners were excused from their primary service to attend the activity. This was done to help individualize the rotation experience according to each student's particular career interests.

### Module design

Each module was designed by the University of Wisconsin Hospital and Clinics PGY 1–3 residents who were either on the combined anatomic/clinical pathology (AP/CP) or AP-only residency track, with mentorship from surgical pathology faculty. The modules were designed using Microsoft PowerPoint and ranged in size from 14 to 46 slides and were assigned to rotating students through the Canvas Learning Management System. Efforts were made to direct learners through a case from the gross to the microscopic findings. Histologic images were heavily annotated, with use of basic PowerPoint animations, to enable students to self-guide through cases without the need for further facilitation. The images utilized in the modules were either sourced from in-house collections or from open-access resources. The modules did not utilize audio or video recordings; however, the Canvas Learning Management System allowed for this functionality, and such recordings could be incorporated at a later date should there be learner requests.

**Fig. 2:**
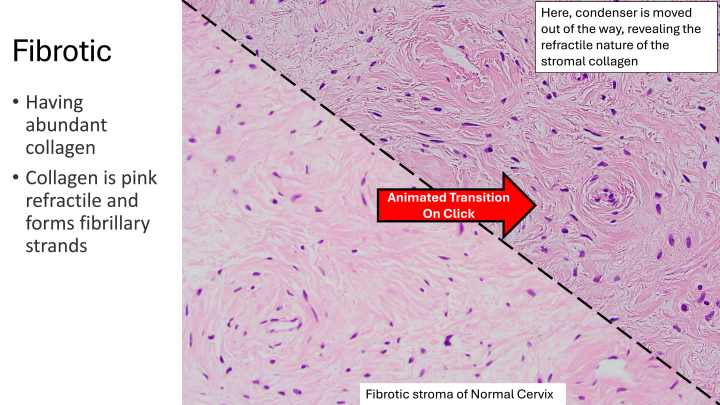
Screenshots of an example slide animation from the basic histopathology modular series. This animation “flips the condenser” on the histologic image upon clicking, to educate the viewer on collagen refractility.

Module content topics included an introductory series on basic histomorphology covering architectural patterns, cellular and cytoplasmic features, stromal features, and nuclear features. Subsequent modules focused on histopathology topics specifically constructed to dovetail with the surgical pathology subspecialty sign-out services that our medical students were most commonly assigned to rotate on (i.e. breast/gynecologic, pediatric, tubular gut, genitourinary, head/neck, endocrine, and hepatobiliary/pancreas). Each of these modules highlighted a different organ system (e.g. kidney, breast, etc.) or relevant disease (e.g. nephroblastoma, ductal carcinoma in situ, etc.), and contained a mixture of educational content presented in bullet points, as well as gross, histologic, and immunohistochemical images of either normal histology or pathology. An example slide animation from the modular content can be seen in Fig. 2.

### Survey design

The effectiveness of the curriculum changes was evaluated via postrotation surveys provided to learners and residents that utilized a combination of a 10-point Likert scale and free-response feedback questions. This novel internal learner survey tool was created by the authors to assess for general rotation experiences, specific module experiences, allow for comparison to anatomic pathology rotations without the new curriculum in place, and provide open-ended feedback. The resident survey tool was designed to assess the new curriculum via subjective perceptions of workload balance with learner education, increased learner histology knowledge, and the importance of opportunities to teach others. All surveys were voluntary without incentives for completion.

Learner surveys were sent via email in the week following completion of their rotation. Resident surveys were sent via email to all pathology residents. Residents who self-identified as having an assigned learner who utilized the new curriculum were asked to complete the survey. All survey data were deidentified and aggregated. Faculty member representatives from each surgical pathology subspecialty as well as the autopsy service were also surveyed utilizing 10-point Likert scale questions. Specifically, they were queried regarding their impressions of medical student baseline histology knowledge and the perceived utility of the new AP medical student curriculum.

### Data analysis

Descriptive metrics for numerical survey questions, mean, range, and sample size (n), were analyzed using Microsoft Excel and GraphPad Prism. Before-and-after survey questions were analyzed for significant differences using a paired *t*-test (GraphPad Prism). Common themes were identified by the authors across free-response feedback survey questions addressing modular content and the overall rotation experience. These themes were assigned thematic codes and summarized. In addition, any learner who happened to be assigned one week on an anatomic pathology service without and with the new curriculum in place was asked to directly compare their experiences between the two weeks. Such rotation assignments were not prearranged but rather of an ad hoc nature.

## Results

### Survey demographics

**Fig. 3:**
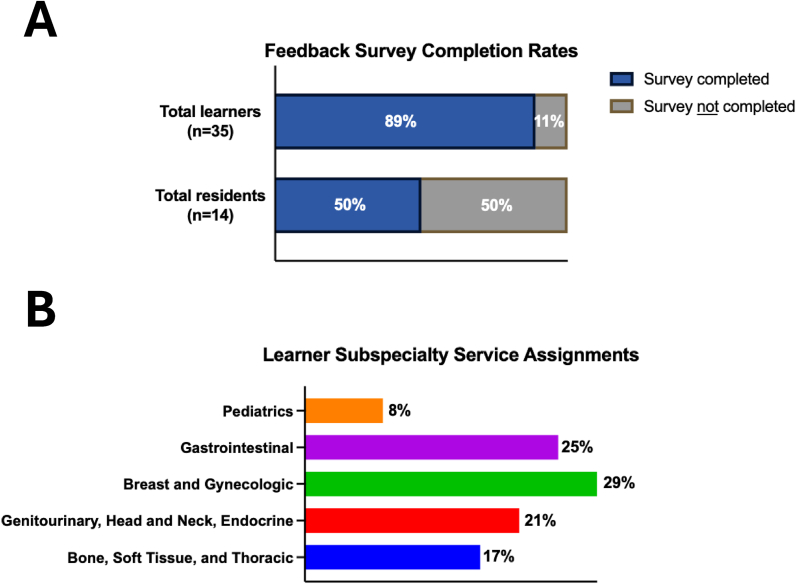
Demographic data of the new curriculum participants, including (A) feedback survey completion rates and (B) learner subspecialty service assignments.

A total of 35 in-house and visiting medical students and the 14 residents they rotated with, to date, have participated in the re-envisioned rotation. All learners were of senior medical student status (i.e. year M3-4) on elective rotations. The survey response rate among medical students was high (31/35; 88.6% response rate), with a lesser response among the residents (7/14; 50.0% response rate). The curricular model was initially piloted on the breast and gynecologic service, with survey comparisons being made to the other surgical pathology services on the traditional curriculum. The bone, soft tissue, and thoracic (BST) modular content had a much longer production time than the other services. This allowed BST to be continually utilized as a control service which remained on the traditional curriculum for comparison purposes after the rollout of content for the remaining services. Demographic data, including the learners’ subspecialty assignment, are visualized in Fig. 3. A total of ten surveys were received by faculty who each supervised multiple student observers over the course of the 2024–2025 academic year and jointly represented each of our surgical pathology subspecialty services as well as the autopsy service.

### Quantitative survey questions and learner knowledge assessments

On a 10-point Likert scale, with 1 corresponding to “poor” or “not helpful” and 10 corresponding to “excellent” or “very helpful,” the organization of the rotation was rated 8.84 (6–10, n = 31). The usefulness of the rotation was rated 8.94 (5–10, n = 31). Student perceptions of their understanding of histology before the rotation were relatively low, i.e. 3.39 (1–6, n = 31), and after the rotation this rose to 6.90 (5–9, n = 31); this difference was statistically significant (*P* < 0.001). Student level of interest in pathology before the rotation was relatively low, i.e. 5.84 (1–10, n = 25), and after the rotation it rose to 8.00 (5–10, n = 31); this difference was statistically significant (*P* < 0.001). Overall experience with the rotation was rated 9.34 (5–10, n = 29). Experience with the independent modules was rated 9.04 (5–10, n = 27). Usefulness of the module content in other aspects of the rotation was rated 8.58 (4–10, n = 26). Usefulness of the bingo card for streamlining learning was rated 7.33 (4–10, n = 9). When asked to compare the experience of rotating on a service which had the modular curriculum in place vs a service which did not, learners felt the experience was improved with the curriculum, i.e. 7.83 (7–9, n = 6). These data are summarized in Table 1. The overall average score for the postmodule quizzes was 97.8% (range 90.5–100). Learners performed evenly across all quizzes, without significant differences in average scores between subspecialty topics.

**Table 1:** Summary of learner feedback survey questions and ratings.

Survey question	Mean rating (range), n
On a scale from 1 (poor) to 10 (excellent), please rate how organized this rotation felt to you.	8.84 (6–10),n = 31
On a scale from 1 (poor) to 10 (excellent), please rate how useful this rotation felt to you.	8.94 (5–10),n = 31
On a scale from 1 (poor) to 10 (excellent), please rate your understanding of histology before this rotation.	3.39^a^ (1–6), n = 31
On a scale from 1 (poor) to 10 (excellent), please rate your understanding of histology after this rotation.	6.90^a^ (5–9), n = 31
On a scale from 1 (very low) to 10 (very high), please rate your level of interest in pathology before this rotation.	5.84^a^ (1–10), n = 31
On a scale from 1 (very low) to 10 (very high), please rate your level of interest in pathology after this rotation.	8.00^a^ (5–10), n = 31
On a scale from 1 (poor) to 10 (excellent), please rate your overall experience with the rotation.	9.34 (7–10), n = 29
On a scale from 1 (poor) to 10 (excellent), please rate your experience with the independent modules.	9.04 (5–10), n = 27
On a scale from 1 (not helpful) to 10 (very helpful), how much did the module content help with other aspects of your rotation?	8.58 (4–10), n = 26
On a scale from 1 (not helpful) to 10 (very helpful), how much did the Bingo card help with streamlining your service learning?	7.33 (4–10), n = 9
On a scale from 1 (far worse) to 10 (far better), please compare your experience between the service with the curriculum and the service without one.	7.83 (7–9), n = 6

a Paired T-test used to compare ratings to “before-and-after” questions (*P* < 0.005).

For the resident survey, the ability to balance learner educational experiences with daily workloads using the new curriculum was improved, i.e. 7.71 (5–10, n = 7). Changes noticed in the baseline understanding of learners since the implementation of the new curriculum were also improved, i.e. 6.86 (5–8, n = 7). The importance of being offered departmental opportunities as residents for involvement in the education of learners was perceived as very important, i.e. 8.29 (6–10, n = 7). These data are summarized in Table 2. For the faculty survey, the perception of rotating medical students’ baseline histology knowledge was low, i.e. 3.5 (1–8, n = 10), and the perception of the utility of the revised AP medical student curriculum was very high, i.e. 9.1 (8–10, n = 10).

**Table 2:** Summary of resident and faculty feedback survey questions and ratings.

Resident survey questions	Mean rating (range), n
On a scale from 1 (far harder to balance) to 10 (far easier to balance), with a 5 indicating no change, how has the implementation of the new learner surgical pathology curriculum changed your ability to balance the educational experiences of your learners with your daily workloads while on AP rotations?	7.71 (5–10), n = 7
On a scale from 1 (even more confused/less satisfied) to 10 (improved baseline knowledge/improved satisfaction), with a 5 indicating no change, what changes, if any, have you noticed in the baseline histology knowledge learners bring to preview and sign-out since the implementation of the surgical pathology curriculum?	6.86 (5–8), n = 7
On a scale from 1 (not important) to 10 (very important), with a 5 indicating indifferent, how important to you is it that the department offers opportunities for residents to be involved in the education of other learners (i.e. medical students, undergraduates, international medical graduates, etc.)?	8.29 (6–10), n = 7

Faculty survey questions	Mean rating (range), n

On a scale from 1 (lacks even basic histology knowledge) to 10 (has far advanced histology knowledge), what is your perception of rotating medical students’ histology skills?	3.5 (1–8), n = 10
On a scale from 1 (not at all useful) to 10 (marked usefulness), how do you perceive the utility of the revised medical student anatomic pathology rotation structure?	9.1 (8–10), n = 10

AP: anatomic pathology.

### Free-response survey questions

Twenty-three free-response comments providing feedback on the modular content were received. Common themes identified for *positive* feedback of the modular content included: overall value of modules for the pathology rotation (16/23; 69.6%), length and organization of modules (8/23; 34.8%), clear and easy-to-understand content (7/23; 30.4%), relevant and useful for clinical practice (7/23; 30.4%), effective quizzes and knowledge checks (7/23; 30.4%), helpful for slide review (5/23; 21.7%), high-quality visuals (4/23; 17.4%), comprehensive overview of topics (3/23; 13.0%), and interactive and engaging elements (2/23; 8.7%). Common themes identified for *constructive* feedback on the modular content included: inclusion of more intermediate-level content (5/23; 21.7 %), more content on immunohistochemistry (4/23; 17.4%), addition of more specialty-specific modules (3/23; 13.0%), incorporation of more diagrams on pathophysiology (3/23; 13.0%), more practice questions (3/23; 13.0%), desire for more interactive learning (2/23; 8.7%), and additional context for high-yield findings (2/23; 8.7%). These data are summarized in Table 3.

**Table 3:** Learner themes among free-response feedback for modular content.

Positive feedback themes	N (% of 23 total)
Overall value of modules for the pathology rotation	16 (69.6)
Length and organization of modules	8 (34.8)
Clear and easy-to-understand content	7 (30.4)
Relevant and useful for clinical practice	7 (30.4)
Effective quizzes and knowledge checks	7 (30.4)
Helpful for slide review	5 (21.7)
High-quality visuals	4 (17.4)
Comprehensive overview of topics	3 (13.0)
Interactive and engaging elements	2 (8.7)

**Constructive feedback themes**	

Inclusion of more intermediate-level content	5 (21.7)
More content on immunohistochemistry	4 (17.4)
Addition of more specialty-specific modules	3 (13.0)
Incorporation of more diagrams on pathophysiology	3 (13.0)
More practice questions	3 (13.0)
Desire for more interactive learning	2 (8.7)
Additional context for high-yield findings	2 (8.7)

Twenty-seven free-response comments providing feedback on the overall rotation experience were received. Common themes identified for *positive* feedback of the overall rotation included: welcoming and supportive learning environment (17/27; 63.0%), high educational value of the rotation (14/27; 51.9%), well-organized rotation structure (8/27; 29.6%), effective use of modules for learning (6/27; 22.2%), appreciation for tailored learning (6/27; 22.2%), appreciation for hands-on learning and practice (6/27; 22.2%), exposure to diverse pathology subspecialties (3/27; 11.1%), and the bingo card for helpful for learning (2/27; 7.4%). Common themes identified for *constructive* feedback on the overall rotation experience included: scheduling and organizational clarity (6 comments, 22.2%), desire for expanded content or learning resources (4 comments, 14.8%), more hands-on experience with grossing (3 comments, 11.1%), request for content on microscope use (3 comments, 11.1%), more practice questions (3 comments, 11.1%), more case-based learning resources (2 comments, 8.7%), and streamlining prerotation resources (2 comments, 8.7%). These data are summarized in Table 4.

**Table 4:** Learner themes among free-response feedback for overall rotation experience.

Positive feedback themes	N (% of 27 total)
Welcoming and supportive learning environment	17 (63.0)
High educational value of the rotation	14 (51.9)
Well-organized rotation structure	8 (29.6)
Effective use of modules for learning	6 (22.2)
Appreciation for tailored learning	6 (22.2)
Appreciation for hands-on learning and practice	6 (22.2)
Exposure to diverse pathology subspecialties	3 (11.1)
Bingo card is helpful for learning	2 (7.4)

**Constructive feedback themes**	

Scheduling and organizational clarity	6 (22.2)
Desire for expanded content or learning resources	4 (14.8)
More hands-on experience with grossing	3 (11.1)
Request for content on microscope use	3 (11.1)
More practice questions	3 (11.1)
More case-based learning resources	2 (7.4)
Streamlining prerotation resources	2 (7.4)

Six free response comments providing feedback on comparison between services with and without the curriculum in place were received. Common themes identified for *positive* feedback of curricular comparison included effectiveness of independent modular learning (6/6; 100%), improved preparedness for interdisciplinary collaboration (3/6; 50.0%), improved baseline confidence in pathology (2/6; 33.3%), customization and flexibility in learning (2/6; 33.3%), enhanced familiarity with common diseases (2/6; 33.3%). Common themes identified for *constructive* feedback of curricular comparison included: difficulty with certain areas due to lack of modular representation (3/6; 50.0%), variation in teaching styles (2/6; 33.3%), request for more case review opportunities (1/6; 16.7%). These data are summarized in Table 5.

**Table 5:** Learner themes among free-response feedback for curricular comparison.

Positive feedback themes	N (% of 6 total)
Effectiveness of independent modular learning	6 (100.0)
Improved preparedness for interdisciplinary collaboration	3 (50.0)
Improved baseline confidence in pathology	2 (33.3)
Customization and flexibility in learning	2 (33.3)
Enhanced familiarity with common diseases	2 (33.3)

**Constructive feedback themes**	

Difficulty with certain areas due to lack of modular representation	3 (50.0)
Variation in teaching styles	2 (33.3)
Request for more case review opportunities	1 (16.7)

## Discussion

The decision to re-envision our traditional medical student rotation structure was prompted by many factors. As was earlier alluded to, case volumes are rising at institutions across the country, and the caseloads that our residents must preview were becoming progressively more challenging to manage, particularly for newer residents. Even without a learner on service, residents may have difficulty finishing their case preview before signing out with their attending physicians. This challenge is compounded when a learner is present, as significant time must be dedicated to teaching basic histology concepts rather than focusing on the case at hand. While learning how to be an effective teacher is an essential skill–and part of the pathology resident Accreditation Council for Graduate Medical Education (ACGME) milestones,^25^ emphasizing the learning of rotating medical students may come at the expense of preview time, which detracts from the resident's own education. One aim of our re-envisioned rotation curricular structure was to equip students with sufficient baseline histology knowledge to engage more meaningfully in service work, thus preserving the educational experience for both learner and resident alike.

Another aim was to address issues related to excessive downtime between rotation activities. It is not uncommon to have either times of low service volume (e.g. days on autopsy service with no cases) or predictable time gaps (e.g. between the end of surgical pathology daily sign-out and the subspecialty quality assurance meeting.) With our prior model, it was challenging for residents and faculty alike to fill the low-service/time gaps with an explicit learning activity as this was when they typically completed tasks of low-learning utility for medical students (e.g. answering emails and other administrative/primarily computer-based tasks). The new rotation and curricular structure offered the opportunity to transform this previously unstructured “study time” into engaging, specific, and achievable educational goals. Medical students could now be redirected either to histology module completion and/or to other ongoing activities in the pathology department that were high-yield given the student's career interests. In short, we sought a re-envisioned AP rotation experience that could provide valuable education even during periods of service downtime without the need for overt resident or faculty facilitation.

An additional important factor in the decision to re-envision our medical student AP experience was one specific to surgical pathology, i.e. how to keep learners engaged and involved in surgical pathology despite converting to a three-day cycle. While previously our one-day surgical pathology cycle (i.e. case preview, sign-out, and grossing all performed by the resident in the same day) lent itself well to medical students simply following the resident's schedule, the transition to a three-day cycle (i.e. day 1: biopsy sign-out and big case preview, day 2: big case sign-out, day 3: grossing) introduced new challenges. In the new three-day cycle, residents would be grossing for an entire 8-h day, 1–2 d a week. While exposure to grossing is important for rotating learners, these longer grossing periods would leave learners with limited educational engagement, as grossing is largely an observational activity for medical students at our institution. Further, grossing can require a high level of concentration from the resident, leaving minimal time for discussion. From the faculty perspective, resident grossing days represented valuable time for faculty to catch up on casework; to teach medical students at such times would be low-yield for the learner and was also almost certain to slow faculty turnaround times, which would be problematic from a patient care perspective. Thus, another aim of re-envisioning our medical student anatomic pathology experience was to focus on high-yield, engaging medical student activities, and minimize the low-yield and repetitious.

Initially, we had considered utilizing outside, ready-made modular content, such as the Association for Academic Pathology's Path Elective^26^ or the American Board of Pathology's Histology Primer,^27^ to supplement service work. Ultimately, however, we elected to construct our modules independently. Even though this approach required much greater initial effort, it also offered several unique advantages. Because these modules were constructed in-house, we retained the ability to easily modify, update, or otherwise edit them in response to learner feedback. Disease prevalence can vary widely depending on the patient population a hospital serves. In-house construction of modules allowed us to emphasize the diseases we felt learners were most likely to encounter while on service at our particular institution. Finally, residents played a large role in the construction of the modules. Allowing residents to aid in the construction of these modules afforded them the opportunity to develop skills in curriculum development and design; while these are critical skills for future medical educators, they can be challenging to get experience in as a resident.

Overall, the data suggest the curricular changes were successful in meeting our objectives. We saw a statistically significant increase in subjective ratings regarding histology knowledge from learners (Table 1). Learners achieved consistently high scores on the postmodule quizzes, indicating objectively appropriate knowledge acquisition in addition to their subjective ratings. Given ratings above eight for both experience and helpfulness of the modular content (Table 1), it can be inferred that learners are gaining meaningful educational value for their rotation from this change. This inference is substantiated by the free-response feedback answers. Learners highlighted the value of the modules for clarity of content presentation, providing a comprehensive overview, and relevance to the service-work portion of their rotation. When comparing a weeklong surgical pathology rotation without and with the curricular changes, learners rated the new curriculum higher than the traditional format and primarily emphasized the modular content in their positive feedback (Table 5).

An additional goal of the curriculum was to embrace a fluid rotation model, allowing for more customization of the rotation experience to fit individual interests and goals of our learners. As part of the positive feedback for the overall rotation experience, learners specifically highlighted this tailored learning aspect (Table 3). Further, this customization element was mentioned as a positive when comparing the new and traditional curricular models (Table 5). A week on an anatomic pathology service can be unpredictable in terms of which disease entities students are exposed to and expected to learn from. The number of varying disease entities—each with their own histologic criteria—can prove overwhelming for some students. To make the scope of the learning more standardized and focused on the “must-know” pathologies, we created modified bingo cards for each service. The cards highlighted a handful of high-yield entities every learner should have exposure to after their weeklong rotation. There was no minimum entity requirement for a successful completion of the card. If a student did not see specific entity listed on the card, and therefore not achieving a “bingo!”, residents and faculty were encouraged to discuss those entities using curated resources and example cases. While there was some mixed feedback, the bingo card was generally well received by those who reviewed it (Table 1). The bingo card was not initially a required component of service work but rather a suggested tool for students to hone their learning. Additionally, not all subspecialty service bingo cards were “rolled out” simultaneously. These factors could account for the relatively low number of learners who provided feedback for this curricular component. Given the positive response from learners who utilized the bingo card, we intend to trial it as a requirement going forward, with either resident or faculty members initialing the various card squares to ensure completion of this requirement. When evaluating all free-response feedback questions from learners, most of our educational interventions were specifically emphasized as positive changes: the independent modular content, customized and individualized learning, and the bingo card (Fig. 1).

Malcolm Knowles’ principles of andragogy emphasize the power of self-directed learning and practical application of knowledge for adult learners.^28^ These principles have demonstrated value for medical education.^29^^,^^30^ Using independent modules and a fluid rotation experience, we have shaped our curricular experience to be more in line with this educational philosophy. Overall, the data are clear from the learner feedback that the rotation provided high educational value and a positive experience. The most mentioned positive feedback comment was the welcoming and supportive learning environment. This underscores the power of having enthusiastic and effective educators teaching pathology to learners, a point that is emphasized as a draw for student interest in the field.^31^ This point is reinforced by a statistically significant increase in subject interest in pathology by learners before and after the rotation (Table 1).

Another major goal of this new model was to provide residents with a better balance between managing their daily workloads and their learner's educational experience. It is clearly important to residents that the department offers opportunities for them to be involved in education of others (Table 2). This becomes more challenging if residents are not offered an environment that maximizes their potential as educators while maintaining a balance with daily workloads. As noted previously, educational skills are part of the resident ACGME milestones, but programs need to establish a formal work environment that facilitates the building of these skills. The ability to redirect learners to self-guided activities during long grossing days or other times likely to provide low-yield educational value to learners allowed residents to maintain productivity without sacrificing educational quality for their students. Survey ratings demonstrate subjectively improved resident perception of this balance (Table 2). Further, residents also endorsed noticing improvements in the baseline histology skillset of learners who took part in the curriculum (Table 2). Faculty similarly expressed strong endorsement of the utility of the new curricular model (Table 2). This endorsement was particularly compelling in light of faculty members' relatively low perceptions of students' baseline knowledge in histology (Table 2). These perceptions likely reflect broader challenges associated with the diminished emphasis on dedicated histopathology instruction commonly observed in integrated, organ-system curricula.

While the general feedback was overall very positive, there were a few emerging themes in the constructive feedback. For the modules, the primary request was for additional content that went beyond the basics of histopathology. While the curriculum was mainly established to instill a baseline histology knowledge for students, it is understandable that many learners would like to go above and beyond. The intention is for intermediate-level learning to occur during service work, as residents and faculty can now emphasize more nuances of the cases at hand. Similarly, learners also asked for more practice questions in general. The postmodule quizzes ranged from four to seven questions per module and only tested information that was deemed most critical by department educators. These questions were not composed to be challenging but rather to reinforce basic concepts. One way to address both the call for intermediate content and more questions would be to provide additional, more challenging questions; this could take the form of more two-tiered or more nuanced questions. Another emphasized request was for more content on immunohistochemistry (IHC). In practice, pathologists use many different immunostains in nuanced ways to make diagnoses. It was difficult to determine how much of this content to include in the modules. It was decided to focus on the most frequently used IHCs in individual organ systems, such as myoepithelial markers for determining ductal carcinoma in situ of the breast versus invasive ductal carcinoma, and leave others for service-based learning. One possible way to address this could be the inclusion of a longer list of relevant IHCs on each service to serve as a reference guide for learners.

Constructive feedback for the overall rotation experience was mostly related to requests for better organization and structure. Interestingly, a separate, larger number of comments referenced rotation organization as a positive. Looking into this discrepancy, it was noticed that most of the comments asking for constructive improvements on rotation organization were from the earliest students rotating on the new curriculum. As time went on, and our department became used to the flow of the new curriculum, feedback on structure and organization gradually shifted from a negative to a positive among students. It is suspected that this trend highlights the early growing pains of implementation and subtle improvements as we gained experience with the model. Other areas of constructive feedback stemmed from a desire for expanded learning resources, especially calls for more modular content on rotations that had not yet transitioned to the new curriculum. Given the gradual rollout of content, some services necessarily lagged behind others in terms of corresponding content availability to learners. This turned out to be advantageous for curriculum evaluation as it allowed us to have students directly compare new and traditional AP curricular options. Now that we have a baseline comparison, and students indicate a preference for the new curricular model, this can be amended easily with the completion and release of content into services that currently lack it. In addition, new content can be added to address some of the specifically mentioned learner interests, such as resources on microscope use. For additional resources beyond our modules, we provided learners with a list of outside resources, such as recommended textbooks and other independent modular content (e.g. Histology Primer and Path Elective).

There are limitations to this study. One obvious limitation is the purposeful exclusion of prerotation surveys. This was done in response to learner requests. Our curriculum-evaluation survey was issued in addition to the required survey sent out by the medical school for rotation evaluation. Out of concern for inducing survey fatigue and not receiving sufficiently detailed feedback on our curriculum model, the decision was made to only include a postrotation feedback survey. Another limitation is the use of an unvalidated survey tool for our feedback. Since there was a pressing need for a curricular intervention due to the 3-day cycle implementation, the department did not have enough time and resources to run survey tool validation studies. As a result, it is possible the survey questions could contain some level of unconscious bias as they are currently written or not accurately evaluate the intended items. Some rudimentary inferences can be made based on the agreement between high quiz-score averages compared with increases in self-evaluated histology knowledge postrotation. Without premodule knowledge checks, however, it is not possible to know the baseline knowledge of these learners, which could be a confounding factor for this data comparison.

While 31 surveys are a robust sample size, the numbers become more limited when stratified into specific categories. Our free-response comparison between new and traditional curricula only had six responses. Additionally, only seven resident educators completed surveys. We sought to survey residents who experienced learner education prior to and postimplementation of the novel curriculum as a means of comparison. The final number of residents who met this inclusion criteria was 14. As these surveys were completely voluntary, and our residents have indicated progressive survey fatigue in other settings, it is suspected that the combination of these factors may account for the 50% resident survey completion rate in our study. Though the data trends are compelling with the current number, additional experience with more learners, residents, and faculty could increase the strength of our arguments. Additional feedback data will be continuously collected as more rotators are exposed to this new model. With survey-based evaluations, there is always a concern for selection bias. Those who complete feedback surveys may not adequately represent the overall study population. In this case, however, the majority of rotating learners completed the survey, giving an accurate representation of the 2024–2025 student pathology rotators and mitigating those concerns. Lastly, while we coded for themes in the free-response feedback questions and utilized the content therein to make inferences about the new curricular model, this is not a robust method for analyzing qualitative data and should not be interpreted as a surrogate for a mixed-methods study. While we feel the data tell a story when summarized, robust conclusions on the qualitative evaluation of our model would require interviews or focus groups with established and accepted methods. This could be an area of future study. For other future directions, we plan to address and implement learner constructive feedback as explored above. Additionally, clinical pathology services have expressed interest in this curricular model; we intend to adapt this model for use on those services.

## Conclusion

Implementation of our novel curriculum, which integrates newly created asynchronous modules with service work and promotes a fluid rotation across different pathology subspecialties, was highly rated amongst rotating learners, resident-educators, and faculty for organization, usefulness, histology education, module experience, rotation personalization, and curricular flexibility. This model has been effective and popular with rotating students, residents, and faculty at our institution and could likewise serve as an effective model for others.

## Author contributions

A.P.T. co-designed the new curricular model along with P.S.W. and E.G.B. J.G. helped refine the curricular model and guide medical students through curricular changes. The resident module design teams were staffed by A.P.T., Q.Z., M.S., A.R.M., C.A., L.E., Q.L., K.E., M.C.G., C.B., and C.C., who created and implemented modules for the various subspecialty services. P.S.W., R.S., S.C., A.P., R.H., and J.J.S. served as faculty advisors for the resident module design teams, suggesting improvements and editing content. A.P.T. analyzed feedback data and wrote the manuscript. E.G.B. advised A.P.T. on the manuscript contents and edited the manuscript.

## Footnote

Portions of this project were presented as an abstract (abstract #: 899) at the United States and Canadian Academy of Pathology 114th Annual Meeting in Boston, MA, in March 2025.

## Funding

The article processing fee for this article was funded by an Open Access Award given by the Society of ‘67, which supports the mission of the Association for Academic Pathology to produce the next generation of outstanding investigators and educational scholars in the field of pathology. This award helps to promote the publication of high-quality original scholarship in *Academic Pathology* by authors at an early stage of academic development.

## Declaration of competing interest

The authors declare no financial interests or other conflicts of interest that could affect the content of this manuscript.
